# Diagnostic Significance of Selected Plasma MicroRNAs in Myelodysplastic Syndromes

**DOI:** 10.3390/ijms27052250

**Published:** 2026-02-27

**Authors:** Svilena Atanasova, Trifon Chervenkov, Maria Teneva, Stela Dimitrova, Ilina Micheva

**Affiliations:** 1 Hematology Clinic, University Hospital “St. Marina”, Medical University, 9000 Varna, Bulgaria; stela.dimitrova@mu-varna.bg (S.D.); ilina.micheva@mu-varna.bg (I.M.); 2 Second Department of Internal Disease, ES Hematology, Medical University, 9000 Varna, Bulgaria; 3 Department of Medical Genetics, Medical University, 9000 Varna, Bulgaria; tcher@mu-varna.bg; 4 Laboratory of Clinical Immunology, University Hospital “St. Marina”, 9000 Varna, Bulgaria; maria.teneva@yahoo.com

**Keywords:** myelodysplastic syndromes, microRNAs, biomarkers

## Abstract

Myelodysplastic syndromes (MDSs) are clonal hematopoietic disorders characterized by ineffective hematopoiesis and their diagnosis remains challenging, requiring integration of clinical, morphological, and genetic data. MicroRNAs (miRNAs) have emerged as potential biomarkers in MDS, offering insights into disease mechanisms and patient stratification. This study aimed to evaluate the diagnostic and prognostic significance of five plasma microRNAs (miR-22-3p, miR-144-3p, miR-16-5p, let-7a-5p, and miR-451a) in 40 patients with MDS, diagnosed according to WHO 2016 criteria and stratified by R-IPSS, and ten healthy controls. Plasma miRNA levels were measured by RT-qPCR. Expression profiles were compared between patients and controls, and further assessed in relation to disease subtypes, risk categories, and clinicopathological features. Expression analysis showed that miR-144-3p, miR-16-5p, let-7a-5p, and miR-451a were significantly lower in MDS patients compared to controls. MiR-451a demonstrated the highest diagnostic predictive value (*p* = 0.0022), followed by miR-16-5p (*p* = 0.0055), miR-144-3p (*p* = 0.0074), and let-7a-5p (*p* = 0.0092). Let-7a-5p was higher in MDS with excess blasts and both let-7a-5p and miR-451a were lower in the low-risk R-IPSS group. Strong correlations between miR-16-5p, miR-144-3p, and miR-451a were observed, probably reflecting their function in erythropoiesis. None of the investigated microRNAs showed independent prognostic significance for overall survival. In conclusion, circulating microRNAs, particularly miR-451a and let-7a-5p, show promise as supportive biomarkers that may complement existing diagnostic and risk assessment tools in MDS. Further studies are needed to validate their clinical applicability.

## 1. Introduction

Myelodysplastic syndromes (MDSs) are a heterogeneous group of clonal hematopoietic disorders characterized by ineffective hematopoiesis, peripheral cytopenias, and an increased risk of progression to acute myeloid leukemia (AML). Diagnosing MDS remains a clinical challenge, requiring a complex integration of clinical, morphological, and genetic findings. Many patients with cytopenia show features that mimic MDS without meeting the diagnostic criteria. Factors like germline mutations, other medical conditions, or prior therapies can cause similar changes, complicating the diagnostic process. These challenges underscore the need for repeated, comprehensive evaluations—including bone marrow analysis, flow cytometry, and molecular testing. A better understanding of MDS pathogenesis could help to identify reliable biomarkers, improving diagnostic accuracy and early detection.

MicroRNAs (miRNAs) are small, non-coding RNA molecules, typically 19–25 nucleotides in length, that regulate gene expression by binding to the 3′ untranslated regions of target messenger RNAs (mRNAs), leading to translational repression or degradation. MicroRNAs have emerged as key regulators in the pathogenesis of MDS, influencing essential cellular processes such as differentiation, proliferation, and apoptosis [1]. Dysregulation of miRNA expression has been reported in MDS, with distinct miRNA profiles identified in patients compared to healthy individuals. These alterations in miRNA expression are associated with various disease aspects, including hematopoietic dysfunction, chromosomal abnormalities, and disease progression. Several studies have demonstrated the potential utility of miRNAs as diagnostic and prognostic biomarkers, offering insights into disease stratification and risk assessment. Despite the growing body of evidence supporting the role of miRNAs in MDS, their exact functional implications and clinical applications require further exploration. While most studies focus on bone marrow-derived miRNAs, the potential of circulating miRNAs as minimally invasive biomarkers remains a promising area of research. In our study, we selected five microRNAs with previously suggested involvement in MDS pathophysiology—miR-22-3p, miR-144-3p, miR-16-5p, let-7a-5p and miR-451a. Their roles in key cellular processes and their potential to serve as diagnostic markers make them relevant candidates for further exploration.

MiR-22 is involved in epigenetic regulation through the suppression of TET2 [2], leading to altered DNA methylation and impaired hematopoietic differentiation, with elevated levels correlating with high-risk MDS [3]. MiR-144 and miR-451a, co-located on chromosome 17q11.2, are essential regulators of erythropoiesis, with miR-144-3p modulating oxidative stress response [4] and miR-451a promoting erythroid differentiation [5]. Their dysregulation has been linked to disease progression and cytogenetic abnormalities in MDS [6]. MiR-16 acts as a tumor suppressor by targeting oncogenes such as BCL2 and VEGF, with its downregulation in MDS contributing to increased angiogenesis and impaired apoptosis [7,8]. Let-7a, a key regulator of differentiation and proliferation, is frequently downregulated in MDS, leading to increased RAS-mediated oncogenic signaling and a higher risk of disease progression and AML transformation [9]. Collectively, these microRNAs influence fundamental biological pathways in MDS and hold potential as diagnostic and prognostic biomarkers.

The aim of our study is to evaluate the diagnostic and prognostic potential of these microRNAs by measuring their plasma levels in MDS patients and healthy controls.

## 2. Results

### 2.1. Baseline Characteristics

The patient cohort consisted of 15 women (37.5%) and 25 men (62.5%), while the control group included 4 women (40.0%) and 6 men (60.0%) (Table 1).

The clinico-laboratory characteristics of the patients are summarized in Table 2. For statistical analysis, patients were categorized as low-risk (very low, low, and intermediate risk with R-IPSS ≤ 3.5) and high-risk (intermediate > 3.5, high, and very high risk).

After sampling, patients received supportive care (n = 13), azacitidine (n = 12), erythropoietin (n = 4), observation only (n = 3), lenalidomide (n = 3), luspatercept (n = 2), or intensive chemotherapy (n = 2). Median follow-up was 14 months (IQR 9–22; range 2–99), with 20 deaths recorded during follow-up.

### 2.2. Differences in MicroRNA Levels Between MDS Patients and Healthy Controls

**Figure 1:**
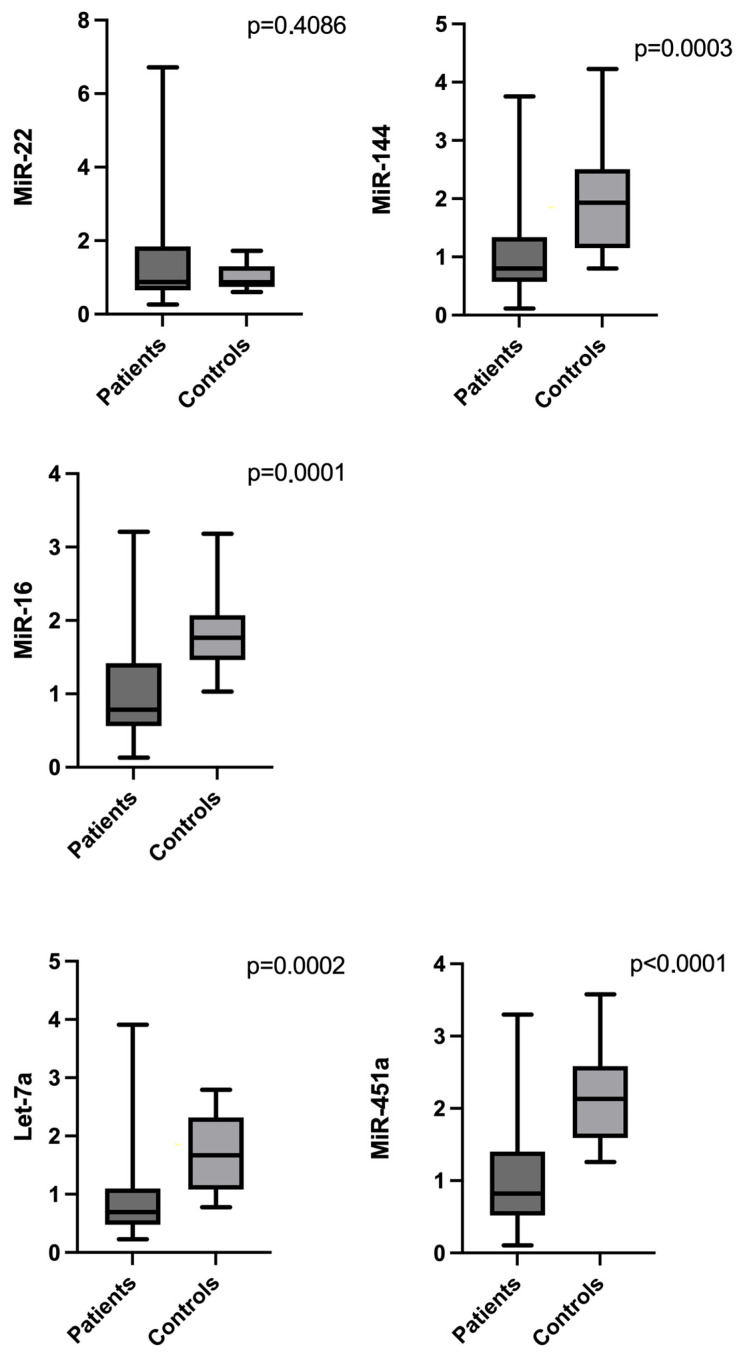
Comparison of plasma levels of the five microRNAs between MDS patients and healthy controls. MicroRNA levels are shown as relative expression calculated by the 2^−ΔΔCt^ method, normalized to cel-miR-39 and expressed relative to a reference sample defined as the arithmetic mean Ct of all individuals. Boxplots show the median (center line) and interquartile range (box); whiskers represent data dispersion as defined in GraphPad Prism. Individual values are provided in Table S1.

Comparative analysis revealed significantly lower expression of miR-144-3p, miR-16-5p, let-7a-5p, and miR-451a in MDS patients compared to healthy controls (*p* < 0.001 for all) (Figure 1). For let-7a-5p, one extreme outlier in the MDS group (defined by the Tukey 3 × IQR criterion) was excluded (n = 39) and the between-group difference remained significant when the value was retained. MiR-451a exhibited the most pronounced difference, with a median expression level of 0.8215 in MDS patients compared to 2.132 in healthy controls, while miR-144-3p (0.802 vs. 1.933), miR-16-5p (0.7855 vs. 1.764), and let-7a-5p (0.692 vs. 1.669) were also lower in the MDS group. In contrast, miR-22-3p expression levels did not differ significantly between the two groups (*p* = 0.4086).

### 2.3. Diagnostic Performance of MicroRNAs: ROC Analysis

**Figure 2:**
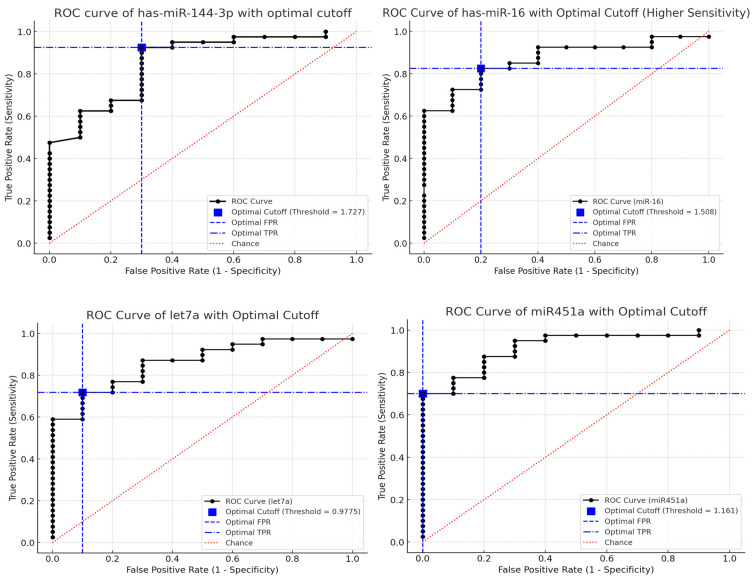
ROC curves for plasma microRNAs distinguishing MDS patients from healthy controls. AUC values with 95% confidence intervals are reported in Table 2. Optimal cutoffs were selected using the Youden index.

ROC analysis confirmed the diagnostic potential of miR-144-3p, miR-16-5p, let-7a-5p, and miR-451a (Figure 2 and Table 3). Among them, miR-451a demonstrated the highest discriminative ability with an AUC of 0.9175, sensitivity 70%, and specificity 100% at the optimal cutoff of 1.161. Optimal cutoffs were determined using the Youden index. MiR-22-3p did not differ significantly between groups in univariate analysis.

### 2.4. Comparison of MicroRNA Levels Among MDS Subgroups

To assess the clinical relevance of the studied microRNAs, their expression levels were compared across different MDS subgroups based on disease subtype, risk stratification, and cytogenetic profile (Table 4). For statistical reliability given the limited sample size, MDS patients were divided into two groups: MDS with low blasts (<5%) and MDS with excess blasts (5–19%). The analysis revealed that let-7a-5p demonstrated a statistically significant difference between the two groups. No statistically significant differences were observed for miR-22-3p, miR-144-3p, miR-16-5p, and miR-451a. However, miR-144-3p (r = −0.319) and miR-16-5p (r = −0.308) exhibited moderate effect sizes, indicating potential differences that may reach significance with a larger cohort.

When stratified according to R-IPSS risk categories (low-risk vs. high-risk), significant differences were observed in let-7a-5p and miR-451a levels. Both were significantly lower in the low-risk group, with large effect sizes. While miR-22-3p, miR-144-3p, and miR-16-5p did not show statistically significant differences, their effect sizes suggest moderate to strong associations, supporting the possibility of meaningful differences in larger samples.

For cytogenetic risk analysis, only patients with intermediate and good cytogenetic risk were included, due to insufficient cases in the very good, poor, and very poor categories. Significant differences were found in miR-144-3p and miR-451a levels, with large and moderate-to-large effect sizes, respectively. 

### 2.5. Correlation Analysis of MicroRNAs

To further explore the role of the selected microRNAs, Spearman correlation analysis was performed. Statistically significant positive correlations were observed among studied microRNAs. Strong correlations were found between miR-22-3p and miR-144-3p (r = 0.7870, *p* < 0.0001), miR-16-5p and miR-451a (r = 0.9559, *p* < 0.0001), and miR-144-3p and miR-451a (r = 0.8860, *p* < 0.0001). Very strong correlations were identified between miR-16-5p and miR-144-3p (r = 0.9246, *p* < 0.0001), and between miR-22-3p and miR-16-5p (r = 0.8336, *p* < 0.0001).

Correlation analysis between microRNA levels and laboratory parameters—including hemoglobin, reticulocytes, leukocytes, neutrophils, platelets, LDH, beta2-microglobulin, ferritin, and erythropoietin—demonstrated several significant associations. MiR-144-3p showed a moderate correlation with LDH (r = 0.3704, *p* = 0.0186) and ferritin (r = 0.3282, *p* = 0.0443), while miR-16-5p (r = 0.3374, *p* = 0.0383) and miR-451a (r = 0.3730, *p* = 0.0211) also correlated significantly with ferritin. Let-7a-5p (r = 0.5652, *p* = 0.0002) and miR-22-3p (r = 0.3970, *p* = 0.0112) showed significant correlations with LDH. No significant correlations were found between microRNA levels and hemoglobin, reticulocytes, leukocytes, neutrophils, platelets, or beta2-microglobulin. Additionally, analysis of microRNA levels in relation to bone marrow blast percentage revealed a statistically significant moderate positive correlation with let-7a. These correlations were explored in a hypothesis-generating manner and should be interpreted cautiously given multiple testing and the limited sample size.

To evaluate potential demographic effects, we also assessed associations between miRNA expression and age and sex in the combined cohort (patients and controls). No significant differences in miRNA levels were observed between males and females (*p* > 0.8 for all). Age showed weak negative associations with miRNA expression overall. A significant inverse correlation was observed only for let-7a-5p (Spearman r = −0.401, *p* = 0.0039).

### 2.6. Predictive Diagnostic Value of MicroRNAs

To further evaluate the potential clinical utility of the studied microRNAs as diagnostic biomarkers, univariate logistic regression analysis was performed. Higher levels of all four microRNAs were associated with a reduced likelihood of having MDS diagnosis. MiR-451a demonstrated the highest predictive value, with an odds ratio (OR) of 0.08977 (95% CI: 0.01317–0.3182, *p* = 0.0022), followed by miR-16-5p (OR = 0.1436, *p* = 0.0055), miR-144-3p (OR = 0.2111, *p* = 0.0074), and let-7a-5p (OR = 0.2393, *p* = 0.0092). Notably, miR-451a had the highest predictive power, explaining approximately 42% of the variation in the diagnostic model (Pseudo-R^2^ = 0.4193).

Given strong intercorrelations among the studied miRNAs, multicollinearity was anticipated in multivariable models. Therefore, LASSO regression was applied for variable selection and identified miR-22-3p and miR-451a as the most significant predictors. Despite miR-22-3p not showing significant differences between MDS patients and healthy controls in earlier analyses, it contributed meaningfully to the diagnostic model. MiR-22-3p showed a positive association with MDS risk (OR = 1.297, 95% CI: 1.100–1.521), while miR-451a maintained its inverse relationship (OR = 0.696, 95% CI: 0.411–1.054). A multivariable logistic regression incorporating both markers confirmed their strong combined predictive power, with the final model achieving an AUC of 0.995 and an overall diagnostic accuracy of 96%. Given the limited sample size and lack of external validation, these model performance estimates should be considered preliminary and require confirmation in independent cohorts.

### 2.7. Predictive Value of MicroRNAs for MDS Subtypes and Risk Groups

The potential of the studied microRNAs to differentiate between MDS subtypes based on blast percentage was evaluated using LASSO regression analysis. Let-7a-5p emerged as the most significant predictor, with an odds ratio (OR) of 4.70 (95% CI: [1.03, 21.41]). This finding was supported by univariate logistic regression, which yielded an OR of 5.943 (95% CI: 1.489–39.69; *p* = 0.0329), and an AUC of 0.722, indicating moderate discriminative ability.

For differentiating high- and low-risk MDS based on R-IPSS, LASSO regression again identified let-7a-5p as the most significant predictor, with an OR of 3.579 (95% CI: [1.000, 57.461]). Univariate logistic regression analysis confirmed this result, with an OR of 8.620 (95% CI: [1.505, 103.1], *p* = 0.0447).

### 2.8. Prognostic Significance of MicroRNAs

The potential prognostic value of the studied microRNAs regarding overall survival (OS) in MDS patients was evaluated using Cox proportional hazards regression analysis. Each microRNA was assessed individually to determine its impact on survival outcomes. The analysis revealed that none of the microRNAs had a statistically significant association with survival. Although the hazard ratios (HRs) for all microRNAs were greater than 1—suggesting a possible trend toward increased mortality risk with higher expression levels—their confidence intervals included 1, confirming the absence of statistical significance (Table 5). The direction of association within the MDS cohort does not necessarily mirror the case–control differences observed at diagnosis and may reflect limited power, clinical heterogeneity, and post-baseline treatment variation.

## 3. Discussion

Epigenetic mechanisms, such as dysregulated DNA methylation, histone modifications, and non-coding RNA expression, play a crucial role in the development of MDS. Among the key epigenetic regulators, microRNAs have emerged as important contributors to MDS pathophysiology by modulating gene expression through translational repression or mRNA degradation.

In this study, we evaluated five circulating plasma microRNAs in an MDS cohort sampled at diagnosis, prior to initiation of therapy, and assessed their diagnostic, subgroup-related, and prognostic relevance. Our findings revealed significant differences in the expression levels of miR-144-3p, miR-16-5p, let-7a-5p, and miR-451a, all of which were downregulated in MDS patients compared to healthy controls, whereas miR-22-3p did not show any significant difference in plasma levels between the two groups. ROC analysis supported the diagnostic potential of the four downregulated miRNAs, with miR-451a showing the strongest discrimination. Within the MDS cohort, let-7a-5p showed the clearest association with disease biology by differentiating blast-defined subgroups and risk categories.

Published data on plasma microRNAs in MDS remain limited and sometimes conflicting, likely reflecting differences in cohort composition, disease spectrum, sample source, and analytical platforms. Ma et al. have reported increased plasma levels of miR-22 in MDS patients, particularly in high-risk cases [3]. A study by Zuo et al. has reported elevated miR-144 levels, particularly in patients with high-risk cytogenetic abnormalities [6]. These discrepancies may, in part, reflect differences in patient selection criteria, as the Zuo et al. study focused on patients with normal cytogenetics with only a small number of cases with single abnormalities of del(7q)/-7 or del(20q). Methodological variations, including differences in detection techniques (RT-qPCR vs. NanoString), may have further influenced the results.

In the case of miR-16-5p, our findings were consistent with those of Zuo et al., who also reported significantly lower plasma levels of miR-16 in MDS patients compared to healthy controls [10]. However, in contrast to our data, which did not show significant differences among MDS subtypes and risk groups, Xiong et al. reported significantly lower miR-16 levels in high-risk patients compared to low-risk ones using bone marrow-derived CD34^+^ cells [7].

Let-7a-5p also emerged as a promising biomarker in our study, with significantly lower expression levels in MDS patients and clear differentiation between subtypes and risk groups. In agreement with our findings, Zuo et al. also reported lower let-7a levels in plasma samples of MDS patients [10]. However, a study by Vasilatou et al. using bone marrow CD34^+^ cells found different trends, reporting lower let-7a levels in high-risk MDS patients compared to low-risk ones [9]. These differences highlight the importance of sample source, as plasma microRNAs may reflect systemic disease processes, while bone marrow cells provide information more directly related to local disease pathophysiology, underscoring the need for caution when comparing studies using different biological materials. Additionally, cellular selection mechanisms, such as the active release of microRNAs into circulation, may contribute to these variations and influence their utility as biomarkers [11].

MiR-451a levels were significantly lower in MDS than in healthy controls. Among patients, miR-451a was lower in the low-risk than the high-risk R-IPSS group, with no differences across disease subtypes. These data support an overall reduction in miR-451a in MDS but show a risk-related pattern that contrasts with Merkerova et al., who reported a greater decrease in high-risk cases [12], and differ from Zuo et al., who observed higher plasma miR-451a in MDS—particularly with adverse cytogenetics such as del(7q)/−7 [6].

Analysis of the five selected microRNAs revealed strong correlations among miR-16-5p, miR-144-3p, and miR-451a, likely reflecting their central roles in erythropoiesis. MiR-16 regulates ribosomal biogenesis and the proliferation of erythroid precursors [13], while miR-144 and miR-451a contribute to oxidative stress regulation and erythroid differentiation through the GATA1 transcription factor [5]. Furthermore, miR-144 and miR-451a share a common locus on chromosome 17q11.2, further supporting their strong correlation. Their coordinated expression suggests a synergistic role in maintaining effective erythropoiesis.

Regarding clinical correlates, we did not observe consistent associations between miRNA levels and routine blood counts, similar to previous reports [10,12], suggesting that circulating microRNAs may reflect broader disease biology rather than isolated hematologic indices. Despite the lack of correlations with routine blood counts, moderate positive correlations were identified between miR-22-3p, miR-144-3p, let-7a-5p, and LDH, a marker of cellular metabolism and tissue damage frequently elevated in MDS. The link between miR-22 and LDH may stem from its involvement in apoptosis regulation via the p53 pathway [14], while let-7a and miR-144 influence glycolysis by targeting glucose transporters such as GLUT1 and GLUT12, affecting cellular energy metabolism [15,16].

Additionally, miR-144-3p, miR-16-5p, and miR-451a showed moderate correlations with ferritin levels, an important marker of iron overload commonly seen in MDS patients. These correlations may reflect the role of these microRNAs in erythroid differentiation and iron metabolism.

From a diagnostic modeling perspective, while several single miRNAs showed good performance, multivariable approaches are particularly relevant given strong intercorrelations among markers. Logistic regression and LASSO analysis identified miR-451a and miR-22-3p as the most significant diagnostic biomarkers, with their combined use achieving high accuracy and specificity in distinguishing MDS patients from healthy controls. Although miR-22-3p was not significant in univariate comparisons, it contributed to the LASSO-selected multivariable model when combined with miR-451a, suggesting complementary predictive information.

Let-7a-5p emerged as the strongest predictor for risk stratification, effectively differentiating between high- and low-risk MDS and distinguishing among MDS subtypes. Our findings are consistent with previous studies, which reported reduced let-7a levels in high-risk MDS patients, supporting its potential involvement in disease progression [10]. Nevertheless, further studies are required to validate its clinical utility.

In terms of prognostic significance, we did not identify any of the microRNAs as independent predictors of survival. However, a possible trend toward increased mortality risk with higher expression levels was observed. This likely reflects the limited sample size, emphasizing the need for validation in larger cohorts of patients. Several studies have revealed associations of microRNAs in MDS with OS and progression-free survival. Zuo et al. found that lower plasma levels of miR-16 and let-7a were associated with worse OS and progression-free survival [10], while Merkerova et al. reported that reduced miR-451a levels correlated with poorer outcome [12]. Additionally, Ma et al. demonstrated that increased miR-22 levels were linked to shorter survival [3].

This study is limited by the modest sample size and the clinical heterogeneity inherent to MDS, which reduces statistical power for subgroup and survival analyses. Quantitative hemolysis QC was not performed, and residual hemolysis or subtle red blood cell carryover cannot be fully excluded despite standardized processing within 1 h and double centrifugation, which is relevant for erythroid-enriched miRNAs (miR-451a, miR-16-5p, miR-144-3p). All samples were collected prior to any treatment, but patients subsequently received different therapeutic approaches and supportive care. This variation may have influenced outcomes and may complicate interpretation of prognostic analyses. In addition, several analyses, including correlations with laboratory parameters and estimates of multivariable model performance, should be considered exploratory and require validation in independent cohorts.

## 4. Materials and Methods

A total of 40 patients diagnosed with MDS from the University Hospital “Sveta Marina,” Varna, were enrolled in the study, along with 10 healthy controls. The study was approved by the Research Ethics Committee (REC) at the Medical University of Varna “Prof. Dr. Paraskev Stoyanov” (Approval Code: 129; 6 April 2023). All participants provided written informed consent prior to enrollment. MDS diagnoses were established according to the WHO 2016 classification which was applied consistently for all patients in this cohort, and patients were stratified by risk category using the Revised International Prognostic Scoring System (R-IPSS). Future studies should re-evaluate these findings using the updated WHO 2022 and ICC frameworks. Peripheral blood was collected at diagnosis and prior to initiation of any disease-modifying therapy. Thus, all miRNA measurements represent a baseline, pre-treatment time point. The control group included 10 clinically healthy individuals (≥18 years), frequency-matched to the patient group by age and sex, who provided written informed consent. Controls had no history of hematologic disease or acute infection at the time of sampling.

### 4.1. RNA Extraction and Reverse Transcription

Peripheral blood samples were collected from all participants into EDTA tubes and processed within 1 h of collection. Plasma was obtained by centrifugation at 3000× *g* for 20 min at 4 °C. The plasma supernatant was carefully transferred to a new tube without disturbing the buffy coat and subjected to a second high-speed centrifugation at 25,000× *g* for 10 min at 4 °C to minimize residual cellular and platelet contamination. The final supernatant was aliquoted (500 µL) and stored at −80 °C until RNA isolation. Hemolysis was not quantified with a dedicated assay (e.g., free hemoglobin or miR-451a/miR-23a ratio), and residual confounding cannot be fully excluded. Aliquots were thawed only once for RNA isolation. MicroRNAs were isolated from 200 µL of plasma using the miRNeasy Serum/Plasma Kit (QIAGEN, Hilden, Germany), following the manufacturer’s protocol. For normalization, 3.5 μL of synthetic spike-in control (C. elegans miR-39) was added to each sample. The RNA was eluted in 25 µL RNase-free water. Reverse transcription was performed using the miRCURY LNA RT Kit (QIAGEN), with a reaction mix consisting of 1.0 µL RNA and 9.0 µL master mix, incubated at 42 °C for 60 min, followed by enzyme inactivation at 95 °C for 5 min.

### 4.2. Quantitative PCR Analysis

Quantitative real-time PCR (qPCR) was conducted using the miRCURY LNA SYBR Green PCR Kit (QIAGEN, Hilden, Germany) and pre-designed primers for the selected microRNAs (let-7a-5p, miR-16-5p, miR-22-3p, miR-144-3p, and miR-451a) and the spike-in control (cel-miR-39-3p). The qPCR reaction consisted of 3.0 µL of diluted cDNA and 7.0 µL of master mix in a total volume of 10 µL, performed in triplicate on a 384-well plate. The thermal cycling conditions used were as follows: enzyme activation at 95 °C for 2 min; 40 cycles of 95 °C for 10 s; 56 °C for 60 s with fluorescence detection; and a melting curve analysis to confirm amplification specificity, consisting of an initial denaturation at 95 °C for 15 s, cooling to 60 °C for 60 s, and gradual heating to 95 °C at a rate of +0.05 °C per second with fluorescence detection.

The analysis was performed using the QuantStudio Dx (Applied Biosystems, USA), and the cycle threshold (Ct) values were recorded for each sample. The relative concentration of the analyzed target microRNAs was determined using the ∆∆Ct method [17], with normalization to the reference microRNA C. elegans miR-39, and relative to a reference sample, which was represented by the arithmetic mean Ct value of all individuals. The calculations were performed using Microsoft Office Excel 2016, and the results were presented as a ratio to the reference sample.

### 4.3. Data Analysis

Statistical analysis was performed using IBM SPSS Statistics v.24, GraphPad Prism v.10.4.1, and Python v.3.8, with a significance level set at α = 0.05. Descriptive statistics (means, standard deviations, medians, percentages) were used to summarize the data. The Shapiro–Wilk test indicated non-normal distribution of microRNA levels; thus, the non-parametric Mann–Whitney U test was applied for comparisons between groups. Extreme outliers were defined using the Tukey criterion (values outside Q1 − 3 × IQR or Q3 + 3 × IQR). One extreme outlier was identified for let-7a-5p in the MDS group and was excluded from the primary comparison. Sensitivity analysis including this value yielded the same conclusion. Receiver Operating Characteristic (ROC) analysis evaluated diagnostic accuracy, using AUC and Youden’s index to define optimal cutoffs. LASSO regression was applied for variable selection and to reduce multicollinearity. Spearman’s correlation assessed relationships between continuous variables, categorized by strength. Simple linear regression was used to evaluate associations (R^2^ as explained variance), logistic regression to assess binary outcomes, and Cox regression to analyze time-to-event data using hazard ratios (HR). Given multiple comparisons, results should be interpreted cautiously and considered exploratory.

## 5. Conclusions

Our analysis indicates that circulating plasma microRNAs are promising non-invasive biomarkers in MDS. We observed consistently lower levels of miR-144-3p, miR-16-5p, let-7a-5p, and miR-451a in patients versus controls, with high diagnostic potential. Let-7a-5p showed subtype- and risk-related variation—higher in excess-blast MDS, and, together with miR-451a, lower in lower-risk R-IPSS. Although none of the microRNAs demonstrated clear prognostic value, observed trends suggest that larger, multi-center investigations are needed to validate these findings and explore the clinical utility of microRNAs in MDS diagnosis, risk stratification, and personalized treatment strategies.

## Figures and Tables

**Table 1:** Baseline demographic characteristics of the study groups (MDS vs. healthy controls). Values are presented as median (interquartile range, IQR) or counts. * Age was compared using the Mann–Whitney U test (two-sided). † Sex distribution was compared using Fisher’s exact test (two-sided).

Variable	MDS (n = 40)	Controls (n = 10)	*p*-Value
Age, years, median (IQR)	71 (67–76)	73 (64–80)	0.799 *
Sex (male/female), n	25/15	6/4	1.000 †

**Table 2:** Clinical and laboratory characteristics of the MDS cohort.

Characteristics	N(%)/Mean
WHO classification	
MDS with multilineage dysplasia	19 (47.5)
MDS with ring sideroblasts	4 (10)
MDS with del(5q)	4 (10)
MDS excess blasts	13 (32.5)
R-IPSS	
Very low	2 (5)
Low	10 (25)
Intermediate	11 (27.5)
High	9 (22.5)
Very high	8 (20)
Cytogenetic risk	
Very good	1 (2.5)
Good	22 (55)
Intermediate	11 (27.5)
Poor	3 (7.5)
Very poor	1 (2.5)
Unknown	2 (5)
Marrow blasts	
<5%	26 (65)
5–9%	2 (5)
10–19%	12 (30)
Hemoglobin (g/L)	79.43 ± 17.52 (28–115)
Reticulocytes (%)	2.495 ± 2.113 (0.32–9.06)
Leucocytes (×10^9^/L)	4.01 ± 2.188 (1.54–10.32)
Neutrophils (×10^9^/L)	2.012 ± 1.553 (0.18–7.79)
Platelets (×10^9^/L)	170.8 ± 164.3 (15–859)
LDH (U/L)	467.2 ± 329.6 (187–1977)
Beta2-microglobulin (mg/L)	3.413 ± 1.518 (1.4–8.1)
Erythropoietin (U/L)	320.3 ± 286.5 (23.5–751)
Ferritin (μg/L)	840.2 ± 626.7 (11.31–3172)

**Table 3:** Receiver operating characteristic (ROC) analysis of plasma microRNAs distinguishing MDS from healthy controls.

MicroRNA	AUC (95% CI)	Optimal Cutoff	Sensitivity (%)	Specificity (%)
miR-144-3p	0.8538 (0.7274–0.9801)	1.727	92.5	70
miR-16-5p	0.8675 (0.7646–0.9704)	1.508	82.5	80
let-7a-5p	0.8615 (0.7536–0.9695)	0.9775	71.79	90
miR-451a	0.9175 (0.8354–0.996)	1.161	70	100

**Table 4:** Results from the comparative analysis of the levels of the five microRNAs across different MDS subtypes, R-IPSS and cytogenetic risk groups. Effect size r was calculated from the Mann–Whitney U test as r = Z/√N; |r| values of 0.1, 0.3, and 0.5 indicate small, moderate, and large effects.

MicroRNA	MDS Subtypes *p*-Value	MDS Subtypes Effect Size (r)	R-IPSS Risk Groups *p*-Value	R-IPSS Risk Groups Effect Size (r)	Cytogenetic Risk Groups *p*-Value	Cytogenetic Risk Groups Effect Size (r)
miR-22-3p	0.3307	−0.291 (small)	0.1132	−0.49 (moderate)	0.2484	−0.29 (small)
miR-144-3p	0.2793	−0.319 (moderate)	0.096	−0.51 (moderate to large)	0.0287	−0.53 (large)
miR-16-5p	0.2998	−0.308 (moderate)	0.0696	−0.56 (moderate to large)	0.0679	−0.45 (moderate to large)
let-7a-5p	0.025	−0.653 (large)	0.0172	−0.753 (large)	0.8759	−0.046 (none)
miR-451a	0.3602	−0.274 (small)	0.0449	−0.62 (large)	0.0481	−0.48 (moderate to large)

**Table 5:** Univariable Cox proportional hazards models for overall survival in the MDS cohort.

MicroRNA	β	SE	HR	95% CI (HR)	AIC
miR-22-3p	0.1447	0.1344	1.156	0.8485–1.460	108.5
miR-144-3p	0.4848	0.2934	1.624	0.8425–2.726	107.3
miR-16-5p	0.5192	0.3302	1.681	0.8338–3.083	107.3
let-7a-5p	0.3197	0.3015	1.377	0.6854–2.304	103.8
miR-451a	0.5012	0.3308	1.651	0.8126–3.012	107.5

## Data Availability

De-identified individual-level data supporting the findings of this study are available in the Supplementary Materials (Table S1).

## Supplementary Materials

The following supporting information can be downloaded at: https://www.mdpi.com/article/10.3390/ijms27052250/s1.
